# Similar Effects of General and Spinal Anaesthesia on Perioperative Stress Response in Patients Undergoing Haemorrhoidectomy

**DOI:** 10.1155/MI/2006/97257

**Published:** 2006-02-09

**Authors:** Unase Buyukkocak, Osman Caglayan, Cagatay Daphan, Kuzey Aydinuraz, Oral Saygun, Tahsin Kaya, Fatih Agalar

**Affiliations:** ^1^Department of Anaesthesiology and Reanimation, School of Medicine, Kirikkale University, 71100 Kirikkale, Turkey; ^2^Kuyuyazisi Caddesi 23/9, 06010, Etlik, Ankara, Turkey; ^3^Department of Biochemistry, School of Medicine, Kirikkale University, 71100 Kirikkale, Turkey; ^4^Department of General Surgery, School of Medicine, Kirikkale University, 71100 Kirikkale, Turkey

## Abstract

Surgery induces release of neuroendocrine hormones (cortisol), cytokines (interleukin-6: IL-6, tumour necrosis factor-α: TNF-α), acute phase proteins (C-reactive protein: CRP, leptin). We studied the effects of general and spinal anaesthesia on stress response to haemorrhoidectomy. Patients were assigned to general and spinal anaesthesia groups (*n* = 7). Blood samples were drawn before induction and 24 hours after surgery. Perioperative levels of IL-6, TNF-α, CRP, cortisol, and leptin were comparable among the groups. Twenty four hours after surgery, TNF-α and cortisol did not change; IL-6 and CRP increased significantly in all patients. Significant increase in leptin levels was found in patients undergoing spinal anaesthesia. Except for the increase in leptin levels, there was no significant difference related to the effects of general and spinal anaesthesia.

## INTRODUCTION

Cytokines are a heterogeneous group of proteins and mediators of the immune-inflammatory response to injury and infection. Interleukin-6 (IL-6) and tumour necrosis factor-α (TNF-α) are proinflammatory components [1]. IL-6 is produced by lymphoid and nonlymphoid cells and affects regulation of T and B cells, IG secretion, acute phase inflammatory reactions, and haematopoiesis. TNF-α is an enhancer of IL-6 secretion and is produced primarily by activated monocytes and macrophages [2].

Surgical stress induces neuroendocrines hormones (cortisol) and cytokines (TNF-α, IL-6). IL-6 plays a major role in initiating the acute phase protein (APP) reaction. C-reactive protein (CRP) is the main APP which increases during inflammatory stimulus, infection, or physical trauma [3, 4].

Leptin (OB protein), is the adipocyte-derived product of antiobesity gene and its circulating concentrations indirectly reflect body fat stores. Serum cytokines may upregulate leptin levels [3, 5, 6]. Leptin is structurally similar to the granulocyte colony-stimulating factor (G-CSF) from IL-6 cytokine family [3]. Circulating leptin level changes acutely during some stressful conditions and has a role in the cross-talk between adipose tissue and the immune system, hypothalamo-pituitary-adrenal (HPA) axis [7, 8]. Leptin has inhibitory effects of cortisol secretion, long-term regulatory effects in the adrenal, and rapid effects on the hypothalamus. High leptin levels inhibit the response of the HPA axis to acute stress [9–11].

Cytokines are elevated during perioperative period [12]. Surgery induces a systemic immunoendocrine response and stimulation of the HPA axis and sympathetic nervous system. Proinflammatory mediators (TNF-α, IL-6) increase proportional to the extent and severity of surgical injury [13]. Activation of neuroendocrine system is related to cytokine family during and after surgery. Interleukins and TNF stimulate some components of HPA axis and thereby increase glucucorticoids which inhibit cytokine production. Anaesthetic methods may affect the cytokine response to surgery changing nervous and hormone pathways [14].

The addition of the spinal block has an advantage over the use of general anaesthesia alone; reducing the neuroendocrine response surgery [15]. The effects of anaesthesia on stress response to haemorrhoidectomy has not been investigated before. Haemorrhoidectomy was preferred as a standard operation to investigate the effects of general and spinal anaesthesia as the extent of stress response created by the operation itself is not to an extent observed in major operations like laparotomy, where the effect of the type of anaesthesia can be negligible. In this study we compared the effects of spinal or general anaesthesia on IL-6, TNF-α, CRP, cortisol, and leptin levels in patients undergoing haemorrhoidectomy.

## MATERIALS AND METHODS

Fourteen patients undergoing elective haemorrhoidectomy were enrolled in the study. Local Hospital Ethics Committee approval and informed consent were obtained from each patient. Exclusion criteria were congestive heart failure, diabetes mellitus, thyroid pituitary, adrenal, kidney, or liver disease, glucocorticoid medication, hypertension, hormone replacement therapy, malignancy, signs of acute infection or inflammation, pregnancy, obesity, malnutrition, and some conditions related to contraindication for spinal anaesthesia. Patients were assigned to general (*n* = 7) or spinal anaesthesia (*n* = 7) according to medical considerations and their desire, if clinically possible.

Body mass index (BMI) values of the patients enrolled in the study were 26 [19–28], 24 [19–27] (median [range]) in general and spinal anaesthesia groups, respectively, since leptin has good positive correlation with body fat mass and BMI.

In order to avoid the confounding effect of diurnal variation, haemorrhoidectomies performed during morning sessions were included in the study. All patients had Grade III and IV haemorrhoidal disease. None of the patients had thrombosed haemorrhoids. All operations were performed by the same surgeons with standard surgical technique, namely Milligan-Morgan haemorrhoidectomy.

For premedication, diazepam 10 mg and atropine 0.5 mg intramuscularly were used in all patients, 1 hour perioperatively.

Patients were monitored using Datex monitor (Datex Ohmeda Cardiocap/5, Louisville, Colo) which included electrocardiogram, noninvasive arterial blood pressure, and pulse oximetry, during the surgery.

In general anaesthesia group (group-general), anaesthesia was induced with intravenous (iv) thiopental 5–7 mg/kg and fentanyl 0.5 μg/kg. Patients received iv vecuronium bromide 0.1 mg/kg to facilitate endotracheal intubation. After intubation, 50% nitrous oxide in oxygen with 2% sevoflurane was used for maintenance.

In spinal anaesthesia group (group-spinal), patients were placed in lateral decubitus position and a 25-gauge spinal needle was inserted through the L3-4 intervertebral space. Hyperbaric bupivacaine 0.5% 2.5 mL (according to patient's height and weight) was injected into subarachnoid space.

Adverse effects such as nausea, vomiting, hypotension (mean arterial pressure < 60 mmHg), bradycardia (heart rate < 60 beats/min with hypotension), hypertension (mean arterial pressure > 110 mmHg), tachycardia (heart rate > 100 /min), and low saturation levels (SpO_2_ < 90%) were recorded perioperatively.

Venous blood samples were drawn just before induction (perioperative) and 24 hours after the operation (postoperative). Blood samples were collected in apirogen tubes with ethylendiamine tetra-acetic acid. After being centrifuged, plasma was stored at −20°C until assayed.

The CRP level was measured by immunoturbidimetric method with Roche kits, using modular analytics P module (Roche). The cortisol levels were determined by electrochemiluminescence immunoassay method with Roche kits, using E-170 (Roche). IL-6, TNF-α, and leptin were measured by micro ELISA (enzyme-linked immunosorbent assay) method with Biosource kits, using μ quant microplate reader (Biotek). The sensitivity, dynamic range, and accuracy of these tests were as follows: < 8 1–1750, and < 2.8% for cortisol; 0.425, 1–280, and < 4.61% for CRP; < 2, 7.8–500, and < 9.3% for IL-6; < 1.7, 15.6–1000, and < 8.5for TNF-α; < 0.36, 1.56–100, and < 4.6% for leptin, respectively.

Statistical analysis was performed with the SPSS for Windows 11.5 statistical programme. All parameters of two groups were compared with Mann-Whitney U. The changes within the groups were analysed using Wilcoxon Signed Ranks Test. Statistical significance was considered at *P* < .05. The results were given as median [range].

## RESULTS

The demographic data showed no significant differences between the groups (Table 1).

Perioperative IL-6, TNF-α, CRP, cortisol, and leptin levels were comparable among the groups (Table 2).

Twenty four hours after surgery, TNF-α and cortisol levels did not change whereas IL-6 and CRP increased significantly, in all patients (*P* = .043 and *P* = .018 in group-general, and *P* = .018 and *P* = .018 in group-spinal, resp). Leptin levels increased in two groups, but the significant increase was found in patients undergoing spinal anaesthesia (*P* = .018) (Table 2).

When comparing the results within the groups, IL-6 levels showed slight increase and TNF-α levels showed slight decrease in group-spinal, postoperatively. These differences were not statistically significant. All other parameters remained similar ranges in two groups (Table 2).

In group-spinal, hypotension developed in one patient just before the operation and iv ephedrine 5 mg was given and the problem was resolved. In group-general, hypertension and tachycardia were observed in two patients during the operation. These symptoms were corrected with hyperventilation thus increasing depth of anaesthesia.

## DISCUSSION

Neuroendocrine system is activated during and after surgery. Surgical stress-induced release of neuroendocrine hormones (eg, cortisol) and cytokines (eg, IL-6, TNF-α) provoke APP (eg, CRP) synthesis and leptin has been shown itself to be an acute phase reactant [3].

Opioids, etomidate, and benzodiazepins have inhibitory effects on the release of cortisol in patients undergoing surgery [12, 16]. In the early postoperative period, HPA systems are activated and cytokines are early responders to surgical stimulation. TNF-α stimulates the production of IL-1β and IL-1β can stimulate the synthesis of IL-6. IL-1, TNF-α, and IL-6 stimulate different components of HPA axis and increase glucocorticoids which are known to inhibit cytokine production [14, 16].

In our study no significant changes in TNF-α levels after surgery were observed in either group and there were no significant differences between the groups. This observation related to TNF-α, is in accordance with a previous study by Høgevold et al [14] in which patients undergoing total hip replacement surgery under general or regional (combine spinal/epidural) anaesthesia were evaluated for stress response. Significantly lower cortisol levels were found during the operation, in the regional anaesthesia group, but in our study, although lower cortisol levels were observed in spinal anaesthesia group, the difference was not significant.

Total intravenous anaesthesia (TIVA) with propofol and alfentanil are known to suppress IL-6 production in abdominal hysterectomy [17]. Helmy et al [1] investigated cytokine production in response to TIVA and cholecystectomy (open, laparoscopic). In their study, IL-6 and TNF-α increased after open cholecystectomy, but this response was absent in laparoscopic cholecystectomy. TIVA had no significant effect on IL-6 and TNF-α. In another study related to the same surgical technique, inhalation anaesthesia supplemented with fentanyl did not modify IL-6, TNF-α, and cortisol response. They concluded that the variables appeared to be mainly dependent on surgical technique [13]. We observed an increase in IL-6 in both spinal and general anaesthesia after haemorrhoidectomy. There was slight increase in group-spinal when compared with group-general, but this increase was not significant.

Cho et al [7] found that a sudden decrease in leptin levels immediately after gastrectomy and an increase 24 hours after surgery. The decrease was explained by the catecholamine release triggered by surgery, and they postulated that cortisol might have potentiated the increase of leptin by insulin on the first postoperative day. Leptin upregulated proinflammatory cytokines (IL-6). In our study, leptin increased 24 hours after surgery as in this study and IL-6 also increased postoperatively, but neither they nor we found any correlation between leptin and IL-6. The increase of leptin 24 hours after surgery is similar to observations reported by previous studies [6, 7, 12, 17].

A study in patients undergoing cardiac surgery showed that perioperative haemodilution influenced cytokine measurements significantly [18].

Haemorrhoidectomy operations are not accompanied by large fluid shifts and the haemodilution related to fluid therapy in spinal anaesthesia, disappears 24 hours after surgery [19].

A previous study related to the effects of epidural anaesthesia on the neuroendocrine response indicated that perioperative epidural blockade of afferent neural impulses did not attenuate the biochemical mediators of the stress response and has no benefit on clinical outcome [4]. Spinal block reduces the neuroendocrine response to surgery. It acts by blockage of ascending sensory pathways and descending sympathetic efferents, and exertion of more prolonged analgesia [15]. In the present study, slight increase in IL-6 and decrease in TNF-α were observed with spinal blockade. The only significant change was evident in leptin levels when compared with general anaesthesia. Leptin secretion is regulated by the other mediators such as IL-6, TNF-α, and cortisol and we found similar levels of cytokines and cortisol in both groups. When considering the number of the patients of the study groups, this statistical difference is not important clinically.

We could not find any effects of anaesthesia on CRP response to surgery, as mentioned before in our studies investigating the effects of anaesthetic techniques on CRP and albumin, during delivery [19] and circumcision [20].

The anaesthetic agents used in spinal anaesthesia, have minimal effects on operation field due to lower concentration in systemic circulation than the anaesthetic agents used in general anaesthesia. Tissue injury of operation field leads to release of mediators which induce cytokine response. This cytokine production is correlated with tissue injury, but there may be a change in suppression of leukocyte functions. General anaesthetic agents have some effects on cytokine response promoting proinflammatory immune response or suppressing of leukocyte functions [21, 22]. We have no information about the effects of general anaesthetic agents used in our study with doses we applied or duration of haemorrhoidectomy, on human immune response and production of proinflammatory cytokines.

In conclusion, we could not find any significant difference related to the effects of anaesthetic techniques on proinflammatory and acute phase protein response to haemorrhoidectomy. Although there was more increase in leptin levels in spinal anaesthesia group, this difference was not clinically relevant.

## Figures and Tables

**Table 1:** Patients' demographics and duration of operation. Data are median [range].

*n* = 7	General anaesthesia	Spinal anaesthesia

Male/female (*n*)	4/3	3/4
Age (years)	49 [31–59]	34 [24–46]
Body mass index (BMI)	26 [19–28]	24 [19–27]
Duration of operation (min)	55 [30–86]	45 [25–80]

**Table 2:** Interleukin-6 (IL-6) and tumour necrosis factor-α (TNF-α), C reactive protein (CRP), cortisol, and leptin levels of the patients. Data are median [range]. Perioperative (before induction). Postoperative (24 hours after the operation).

*n* = 7	Pre/postoperative	General anaesthesia	Spinal anaesthesia

IL-6 (pg/mL)	Perioperative	1.63 [0.95–11.67]	5.16 [1.22–8.25]
	Postoperative	6.65 [1.76–22.66]^1^	11.94 [7.6–25.51]^2^
TNF-α (pg/mL)	Perioperative	12.48 [6.84–26.66]	6.60 [1.71–34.00]
	Postoperative	9.78 [6.36–26.17]	5.87 [2.20–16.14]
CRP (mg/L)	Perioperative	1.25 [0.54–6.56]	1.64 [0.51–26.06]
	Postoperative	11.34 [1.29–29.29]^2^	6.27 [3.77–58.74]^2^
Cortisol (nmol/L)	Perioperative	346 [182–1613]	377 [80–696]
	Postoperative	351 [218–550]	204 [70–562]
Leptin (ng/mL)	Perioperative	6.15 [2.59–18.44]	4.95 [3.95–26.87]
	Postoperative	6.38 [3.12–35.05]	10.58 [6.96–30.55]^2^

^1^Significantly higher than perioperative levels (*P* = .043).

^2^Significantly higher than perioperative levels (*P* = .018).
